# Supplementary material for: Disentangling the contribution of hospitals and municipalities for understanding patient level differences in one-year mortality risk after hip-fracture: A cross-classified multilevel analysis in Sweden
Source: PLoS One. 2020 Jun 3;15(6):e0234041. doi: 10.1371/journal.pone.0234041 (PMC7269247; doi:10.1371/journal.pone.0234041)
Supplement: S1 Table — (DOCX) [file pone.0234041.s001.docx]

**S1_Table: The logistic regression to obtain a sociodemographic risk score (i.e., individual predicted probability) of one-year mortality as a function of those sociodemographic variables**

|  |  | Odds Ratio | Coef. |
| --- | --- | --- | --- |
|  |  |  |  |
| SOCIO-ECONOMIC Risk score | age | 0.99 (1.05-1.05) | -0,01 |
|  | age^2^ | 1.00 (1.00-1.00) | 0 |
|  |  |  |  |
|  | Gender (male reference) | 2.14 (2.05-2.24) | 0,762 |
|  |  |  |  |
|  | Income Low | Reference | Reference |
|  | Income Middle | 0.96 (0.90-0.99) | -0,06 |
|  | Income High | 0.86 (0.81-0.91) | -0,15 |
|  |  |  |  |
|  | Cohabiting (Yes reference) | 0.86 (0.82-0.90) | -0,15 |
|  |  |  |  |
|  | Swedish native | 0.99 (0.92-1.07) | -0,01 |
|  |  |  |  |
|  | High Education (by family) | 0.89 (0.83-0.95) | -0,12 |
|  |  |  |  |
|  | Cons |  | -4,23 |
|  |  |  |  |
|  | ROC | 0.68 (0.67-0.69) |  |
