# Supplementary material for: Disentangling the contribution of hospitals and municipalities for understanding patient level differences in one-year mortality risk after hip-fracture: A cross-classified multilevel analysis in Sweden
Source: PLoS One. 2020 Jun 3;15(6):e0234041. doi: 10.1371/journal.pone.0234041 (PMC7269247; doi:10.1371/journal.pone.0234041)
Supplement: S2 Table — (DOCX) [file pone.0234041.s002.docx]

**S2_Table: The logistic regression to obtain a biomedical risk score (i.e., individual predicted probability) of one-year mortality as a function of those previous diseases including ICD-10 codes:**

|  |  | Odds Ratio | Coef. |
| --- | --- | --- | --- |
|  |  |  |  |
| Medical risk score | Osteoporosis (M80-M81) | 0.90 (0.83-0.98) | -0,1 |
|  | Chronic kidney disease (N18) | 1.59 (1.45-1.74) | 0,46 |
|  | Acute myocardial infarction (I21) | 1.33 (1.22-1.44) | 0,28 |
|  | Heart failure (I50) | 2.11 (2.00-2.23) | 0,75 |
|  | Other peripheral vascular diseases (I73) | 0.93 (0.79-1.08) | -0,08 |
|  | Cerebrovascular diseases (I60-I69) | 1.20 (1.13-1.27) | 0,18 |
|  | Atherosclerosis- Aortic aneurysm and dissection (I70-I71) | 1.27 (1.15-1.39) | 0,24 |
|  | Dementia (F01-F03) | 2.34 (2.21-2.48) | 0,85 |
|  | Chronic lower respiratory diseases (J40-J47 ) | 1.22 (1.15-1.30) | 0,2 |
|  | Other disorders of the skin and subcutaneous tissue (L80-L99) | 0.85 (0.79-0.92) | -0,16 |
|  | Peptic ulcer (K27) | 1.21 (0.82-1.79) | 0,19 |
|  | Diseases of liver (K70-K77) | 1.47 (1.20-1.81) | 0,39 |
|  | Diabetes mellitus (E08-E13) | 1.00 (0.95-1.06) | 0 |
|  | Hemiplegia (G81) | 0.82 (0.69-0.99) | -0,19 |
|  | Neoplasms (C00-D49) | 1.44 (1.38-1.51) | 0,36 |
|  | Human Immunodeficiency Virus (HIV) (B20) | 1.86 (0.40-8.52) | 0,62 |
|  | Hip fracture (previous)(S70-S72) | 1.18 (1.11-1.25) | 0,16 |
|  | cons |  | -1,52 |
|  | ROC | 0.64 (0.63-0.64) |  |
