# Supplementary material for: Disentangling the contribution of hospitals and municipalities for understanding patient level differences in one-year mortality risk after hip-fracture: A cross-classified multilevel analysis in Sweden
Source: PLoS One. 2020 Jun 3;15(6):e0234041. doi: 10.1371/journal.pone.0234041 (PMC7269247; doi:10.1371/journal.pone.0234041)
Supplement: S1 File — (DOCX) [file pone.0234041.s003.docx]

**S1_file: An extended explanation of the methodology**

**The regression models for one-year mortality:**

The first model (**model 1**) was a single-level logistic regression including socioeconomic risk in four groups:

$$y_{i}\sim\mathrm{Binomial}\left( 1,\pi_{i} \right)$$

$$\mathrm{logit} \left( \pi_{i} \right)\equiv\log\left( \frac{\pi_{i}}{1-\pi_{i}} \right)=\beta_{0}+\beta_{1}{Risk2}_{i}+\beta_{2}{\mathrm{Risk}3}_{i} +\beta_{3}{\mathrm{Risk}4}_{i}$$

where $\pi_{i}$ denotes the probability of death for patient $i$.

The second model (**model 2**) expanded model 1 to include the risk score for medical factors and use of bisfosfonates, analgestics, psycholeptics and psychoanaleptics

$$y_{i}\sim\mathrm{Binomial}\left( 1,\pi_{i} \right)$$

$$\mathrm{logit} \left( \pi_{i} \right)\equiv\log\left( \frac{\pi_{i}}{1-\pi_{i}} \right)=\beta_{0}+\beta_{1}{Risk2}_{i}+\beta_{2}{\mathrm{Risk}3}_{i} +\beta_{3}{\mathrm{Risk}4}_{i}$$

$$+\beta_{4}\mathrm{Bifosfonates}_{i}+\beta_{5}\mathrm{analgestics}_{i}+\beta_{6}\mathrm{psycholeptics}_{i}+\beta_{7}\mathrm{psychoanaleptics}_{i}$$

$$+\beta_{8}\mathrm{RiskMedical}2_{i}+\beta_{9}\mathrm{RiskMedical}3_{i}+\beta_{10}\mathrm{RiskMedical}4_{i}$$

The third model (**model 3**) we expanded model 2 by converting it into a two-way cross-classified multilevel model with the patients nested within the 54 hospitals and the 290 municipalities. For this purpose, we add two random effects, one for the hospital level and the other for municipality level. The model is written as

$$y_{i}\sim\mathrm{Binomial}\left( 1,\pi_{i} \right)$$

$$\mathrm{logit} \left( \pi_{i} \right)\equiv\log\left( \frac{\pi_{i}}{1-\pi_{i}} \right)=\beta_{0}+\beta_{1}{Risk2}_{i}+\beta_{2}{\mathrm{Risk}3}_{i} +\beta_{3}{\mathrm{Risk}4}_{i}$$

$$+\beta_{4}\mathrm{Bifosfonates}_{i}+\beta_{5}\mathrm{analgestics}_{i}+\beta_{6}\mathrm{psycholeptics}_{i}+\beta_{7}\mathrm{psychoanaleptics}_{i}$$

$$+\beta_{8}\mathrm{RiskMedical}2_{i}+\beta_{9}\mathrm{RiskMedical}3_{i}+\beta_{10}\mathrm{RiskMedical}4_{i}$$

$$+v_{k}+u_{j}$$

$$v_{k}\sim N\left( 0,\sigma_{v}^{2} \right)$$

$$u_{j}\sim N\left( 0,\sigma_{u}^{2} \right)$$

where $v_{k}$ and $u_{j}$ denotes the random effects for the hospital and the municipality levels respectively. The random effects are assumed to be normally distributed with mean 0, between-hospital variance$\sigma_{v}^{2}$ and between-municipality variance$\sigma_{u}^{2}$. Thereby it is possible to quantify the hospital and municipality general contextual effect [1]. That is, the share of the total patient variance in the propensity of death that is at the hospital and the municipality levels over and above patients’ characteristics. Information on the general contextual effect allows us to disentangle the contribution of each care setting for understanding patient level differences in vital prognosis.

#### **Calculating the variance partition coefficient**

We calculated the variance partition coefficient (VPC) [2-4], namely the proportion of adjusted individual propensity for hip fracture variation that lies between the hospital/municipality units. These statistics are derived from the latent response formulation of the logistic regression model where the patient-level residuals follow a logistic distribution with a mean of 0 and a constant variance of 3.29 [5]. The formula for the VPC for the hospital level is

$$\mathrm{VPC}_{H}=\frac{\sigma_{v}^{2}}{\sigma_{v}^{2}+\sigma_{u}^{2}+3.29}$$

And for the municipality level

$$\mathrm{VPC}_{M}=\frac{\sigma_{u}^{2}}{\sigma_{v}^{2}+\sigma_{u}^{2}+3.29}$$

The joint VPC of the hospital and municipalities can be calculated as

$$\mathrm{VPC}_{\mathrm{HM}}=\frac{\sigma_{v}^{2}+\sigma_{u}^{2}}{\sigma_{v}^{2}+\sigma_{u}^{2}+3.29}$$

We multiply these VPCs by 100 to express them as percentages.

#### **Using the area under the receiver operator characteristics curve (AUC)**

We calculate the predicted probability for each model and use it to obtain the area under the receiver operator characteristics curve (AUC) [6]. The AUC is constructed by plotting the true positive fraction (i.e., sensitivity) against the false positive fraction (i.e., 1 – specificity) for different binary classification thresholds of the predicted probability for one-year mortality. Thus, the AUC measures the accuracy of the information provided by the model for discriminating individuals who die from those who survive. The AUC takes a value between 0.5 and 1, where 1 indicates perfect discrimination and 0.5 means that the studied variables have no discriminatory accuracy at all. Thereafter we quantified the change in the AUC ( $\Delta$:AUC ) by comparing consecutive models [7].

$$\Delta:AUC ={AUC}_{m+1}-{AUC}_{m}$$

Where $m$ is the initial model and $m+1$ is the extended model.

The higher the $\Delta:AUC$, the larger the improvement of the discriminatory accuracy provided by the information conveyed by the new model. Observe that the prediction from model 3 includes the random effect of the hospital and/or the municipality levels [1, 8]. From this perspective comparing model 3 with model 2 informs on the general contextual effect of the hospital and/or the municipality levels. That is, after adjustment for patient case-mix, the higher the $\Delta$-AUC, the higher the relevance the hospital/municipality context for predicting the vital status of the patients one year after the hospital stay. In model 3, the hospital/municipality random effects embrace all known and unknown contextual factors that condition one-year mortality over and above the known characteristics of the patients.

### **Software and estimation methods**

We ran all models in MLwiN 3.02 [9] called from Stata 14.1 using the runmlwin command [10]. We note that MLwiN can equally be called from within R using the sister R2MLwiN package ([11]) and so our analysis can also be replicated by readers in that statistical package. Estimation was performed using Markov chain Monte Carlo (MCMC) methods (21), with diffuse (vague, flat, or minimally informative) prior distributions for all parameters. We used quasilikelihood methods to provide starting values for all parameters. For each model, the burn-in length was 500 iterations and the monitoring chain was 5,000 iterations. Visual assessments of the parameter chains and standard MCMC convergence diagnostics suggested that these iteration lengths were adequate. We calculated point estimations and uncertainty of the parameters using the chains in the MCMC estimations to obtain the median and the 95% credible intervals. In the complementary material, we provide the Stata do-file that can be used to replicate the analyses.

**References:**

1 Merlo J, Wagner P, Ghith N, et al. An Original Stepwise Multilevel Logistic Regression Analysis of Discriminatory Accuracy: The Case of Neighbourhoods and Health. *PloS one* 2016;11:e0153778.

2 Larsen K, Petersen JH, Budtz-Jorgensen E, et al. Interpreting parameters in the logistic regression model with random effects. *Biometrics* 2000;56:909-14.

3 Larsen K, Merlo J. Appropriate assessment of neighborhood effects on individual health: integrating random and fixed effects in multilevel logistic regression. *Am J Epidemiol* 2005;161:81-8.

4 Merlo J, Chaix B, Ohlsson H, et al. A brief conceptual tutorial of multilevel analysis in social epidemiology: using measures of clustering in multilevel logistic regression to investigate contextual phenomena. *Journal of epidemiology and community health* 2006;60:290-7.

5 Goldstein H, Browne W, Rasbash J. Partitioning variation in multilevel models. *Understanding statistics: statistical issues in psychology, education, and the social sciences* 2002;1:223-31.

6 Pepe MS, Janes H, Longton G, et al. Limitations of the odds ratio in gauging the performance of a diagnostic, prognostic, or screening marker. *Am J Epidemiol* 2004;159:882-90.

7 Merlo J, Wagner P, Ghith N, et al. A novel stepwise multilevel logistic regression approach to analysis of individual heterogeneity using measures of discriminatory accuracy: the case of neighbourhoods and health. 2015.

8 Wagner P, Merlo J. Discriminatory accuracy of a random effect in multilevel logistic regression. *International Journal of Epidemiology* 2014;44:i49-i50.

9 Charlton C, Rasbash J, Browne WJ, et al. MLwiN Version 3.00. Centre for Multilevel Modelling, University of Bristol. 2017.

10 Leckie G, Charlton C. runmlwin - A Program to Run the MLwiN Multilevel Modelling Software from within Stata. . *Journal of Statistical Software* 2013;52:1-40.

11 Zhang Z, Parker RM, Charlton CM, et al. R2MLwiN: A package to run MLwiN from within R. *Journal of Statistical Software* 2016;72:1-43.
