# Supplementary material for: Disentangling the contribution of hospitals and municipalities for understanding patient level differences in one-year mortality risk after hip-fracture: A cross-classified multilevel analysis in Sweden
Source: PLoS One. 2020 Jun 3;15(6):e0234041. doi: 10.1371/journal.pone.0234041 (PMC7269247; doi:10.1371/journal.pone.0234041)
Supplement: Attachment — Submitted filename: Response to Reviewers.docx [file pone.0234041.s004.docx]

**Response to Reviewers**

1. Please ensure that your manuscript meets PLOS ONE's style requirements, including those for file naming. The PLOS ONE style templates can be found at
http://www.journals.plos.org/plosone/s/file?id=wjVg/PLOSOne_formatting_sample_main_body.pdf and http://www.journals.plos.org/plosone/s/file?id=ba62/PLOSOne_formatting_sample_title_authors_affiliations.pdf

**Reply: We thank for pointing out this example of author guidelines. On the title page we have added the ¶ symbol for 1st set of equal contributors. We have corrected the headings to be written in sentence case instead of capitalize. In addition, we have deleted the graphic images and space in the tables.**

2. We note that you have indicated that data from this study are available upon request. PLOS only allows data to be available upon request if there are legal or ethical restrictions on sharing data publicly. For information on unacceptable data access restrictions, please see <http://journals.plos.org/plosone/s/data-availability#loc-unacceptable-data-access-restrictions>. In your revised cover letter, please address the following prompts:

a) If there are ethical or legal restrictions on sharing a de-identified data set, please explain them in detail (e.g., data contain potentially identifying or sensitive patient information) and who has imposed them (e.g., an ethics committee). Please also provide contact information for a data access committee, ethics committee, or other institutional body to which data requests may be sent.

b) If there are no restrictions, please upload the minimal anonymized data set necessary to replicate your study findings as either Supporting Information files or to a stable, public repository and provide us with the relevant URLs, DOIs, or accession numbers. Please see http://www.bmj.com/content/340/bmj.c181.long for guidelines on how to de-identify and prepare clinical data for publication. For a list of acceptable repositories, please see http://journals.plos.org/plosone/s/data-availability#loc-recommended-repositories.

We will update your Data Availability statement on your behalf to reflect the information you provide.

**Reply: We apologize for the lacking information on data approval and access. The dataset are only available from the Swedish National Board of Health and Welfare and Statistics Sweden upon request following an ethical approval. The database was constructed after approval from the Ethical Committee in Sweden (**<https://etikprovningsmyndigheten.se/>**) and from the data safety committees of the Swedish National Board of Health and Welfare (**<https://www.socialstyrelsen.se/>**) and Statistics Sweden (**<https://www.scb.se/en/>**).**

3. Thank you for stating the following in the Acknowledgments Section of your manuscript:
"we thank the Health Research Fund of Central Denmark Region Denmark and the Swedish Research Council for supporting this work https://www.swecris.se/betasearch/details/project/201701321VR and Vetenskapsrådet projekt-id: 2017-01321." We note that you have provided funding information that is not currently declared in your Funding Statement. However, funding information should not appear in the Acknowledgments section or other areas of your manuscript. We will only publish funding information present in the Funding Statement section of the online submission form. Please remove any funding-related text from the manuscript and let us know how you would like to update your Funding Statement. Currently, your Funding Statement reads as follows:
"Juan Merlo
Vetenskapsrådet projekt-id: 2017-01321
https://www.swecris.se/betasearch/details/project/201701321VR
NO

Pia Kjær Kristensen
The Health Research Fund of Central Denmark Region
A869
https://www.rm.dk/sundhed/faginfo/forskning/region-midtjyllands-sundhedsvidenskabelige-forskningsfond/
NO"

**Reply: We thank the editor for pointing this out. We have deleted the phrases regarding funding statement in the acknowledgement section in the manuscript page 19 from line 340 to line 343. The original expression in the acknowledgement section (“the Swedish Research Council for supporting this work** [**https://www.swecris.se/betasearch/details/project/201701321VR**](https://www.swecris.se/betasearch/details/project/201701321VR) **and Vetenskapsrådet projekt-id: 2017-01321”) was unfortunately confusing as it may represent two different funding statements. However, the “Swedish Research Council” is the English name of Swedish “Vetenskapsrådet”. So the funding statement for Juan Merlo is:
Swedish Research Council (i.e., Vetenskapsrådet) project-id: 2017-01321.** [**https://www.swecris.se/betasearch/details/project/201701321VR**](https://www.swecris.se/betasearch/details/project/201701321VR)

**The funders had no role in study design, data collection and analysis, decision to publish, or preparation of the manuscript.**

Reviewer #1: This paper reports an analysis of national data on hip fracture mortality in older adults using a cross-classified multilevel framework to examine potential cluster effects by hospital and by municipality. Strengths of this study include the use of a large and comprehensive dataset and a sophisticated analytical approach. Overall, this is a strong contribution and my comments are relatively minor.

**Reply: We thank the reviewer for the kind remarks.**

1. Additional supplementary information regarding the calculation of the sociodemographic and clinical risk scores would be valuable -- e.g., results for the logistic regression models from which these scores were derived would be good to include in the supplemental materials, including model fit and the parameter estimates used to compute the individual risk scores.
**Reply: We agree with the reviewer that calculation of the sociodemographic and clinical risk scores would be valuable for the interested reader. We have, therefore, as suggested by the reviewer, updated the Statistical appendix (labeled S1-S2 Table) to include information on the beta coefficients for the included variables in the Socioeconomic and clinic risk score equations as well as proving AUC values.**

2. Because the cluster VPC statistics were calculated only after controlling for patient sociodemographic and clinical factors, there is some risk of under-estimating geographic disparities due to clustering of sociodemographic risk factors and patient comorbidity rates. It is likely that these risk factors are themselves clustered by municipality (and potentially to a lesser extent by hospital) based on local deprivation. The analytic reasons for taking the approach you used is explained clearly, but the issue of risk clustering should be briefly addressed in the discussion section. In particular, the suggestion that interventions aimed at improving mortality outcomes should focus on individual factors at a national level rather than on focusing on particular low-performing hospitals and municipalities should be somewhat tempered by the fact that certain specific places may require more focussed intervention because they include clusters of high-risk patients.

**Reply: We thank the reviewer for bringing our attention to the possible risk of under-estimating geographic disparities due to clustering of sociodemographic risk factors and patient comorbidity rates. We have now estimated the VPC statistics for the empty model for considering the maximal clustering. The empty model had a VPC values for both hospitals and municipalities of 0.007. We therefore, think that the risk of under-estimating geographic disparities is minor. Besides, even if the empty VPC were much higher and reduced when considering the composition of patients (because some hospitals include clusters of high-risk patients), the directing resources to high-risk patients on a national basis would automatically allocate more resources to those hospitals and municipalities with more high-risk patients. We have therefore, elaborated the paragraph on page 17 from line 312 to line 318 in the Discussion section to the flowing:**

***We found that hospital and mortality differences in average mortality risks were only a minor share of the total individual variance in the propensity to die or that, in other words, adding the hospital and municipality level does not increase the discriminatory accuracy obtained by patient level information only. This means that the hospital and municipality performances are homogenous overall in the country. Therefore, our results indicate that we will not lower mortality after hip fracture in Sweden by focusing on specific hospitals or specific municipalities with high average mortality. Rather, special efforts to reduce mortality after hip fracture should be focused on vulnerable patient groups of hip fracture patients wherever they are. From this reason, however, hospitals and municipalities with a higher number of vulnerable patients than other will need more intense interventions.***
